# Molecular Research in Human Microbiome

**DOI:** 10.3390/ijms241914975

**Published:** 2023-10-07

**Authors:** Maria Teresa Mascellino

**Affiliations:** Department of Public Health and Infectious Diseases, Sapienza University of Rome, 00185 Rome, Italy; mariateresa.mascellino@uniroma1.it

Recent evidence has shown that the human microbiome is associated with a wide range of diseases, from non-neoplastic to tumourigenesis, including cancer, inflammation, intestinal damage, etc. Thus, alterations in the normal gut integrity are present during *Clostridioides difficile* infections (CDIs) [1,2]. Some studies have also demonstrated a close relationship between gut microbiota metabolism and cerebral stroke [3]. Our microbiota plays a vital role in our health; it protects us against pathogens, promotes the development of our immune system, and helps metabolize various compounds. Maintaining a balanced microbial ecosystem is essential for protecting our health. The application of omics technology to investigate the mechanism underlying the role of gut microbiome is crucial.

The microbiome is shown to be especially involved in cancer. In this situation, most bacteria, such as salivary and fecal microbiome, other than circulating microbial DNA in blood plasma, impact various kinds of therapies (radiotherapy, chemotherapy, and immunotherapy). Kneis et al., 2023, [4] widely underlined the specificity of the microbiome in different parts of the colon (right- or left-sided colon) or in the rectal portion [4]. This situation affects the progression or outcome of cancer. The right- and left-sided colon have distinct embryological origins and different clinical and molecular characteristics. Thus, they harbor distinct niches and have different microbiome compositions. Another study has indicated that the gut microbiota may serve as a potential target in cancer therapy modulation by enhancing the effectiveness of chemotherapy or immunotherapy [5]. In this article, the role of the microbiome in cancer treatment is evaluated, speculating a potential connection between treatment-related microbial changes and cardiotoxicity. The authors investigate some bacterial families of the microbiome and their possible relationship between cancer treatment and cardiac disease. In this case, a serious consequence due to cancer treatment could be avoided, potentially reducing this fatal side effect (cardiotoxicity), focusing on a potential complex interaction among the microbiome, cancer treatment, and cardiovascular diseases.

*Helicobacter pylori* is involved in different ranges of infections, such as gastritis, peptic ulcer, atrophic gastritis, gastric cancer, and gastric MALT-lymphoma [6]. The establishment of a correct therapy is crucial for eradicating *H. pylori* infection. The use of empiric or tailored therapy may depend on several factors, such as concomitant diseases, number of previous antibiotic treatments, differences in bacterial virulence in individuals with positive or negative cultures, together with local antibiotic resistance patterns in real-world settings. The regional knowledge of clarithromycin and levofloxacin resistance is very important to establish an appropriate therapy in different geographical areas.

Metabolomic technologies may provide critical information about the role of gut microbiome in cancer. Liver cancer, liver cirrhosis, and emerging therapies for hepatocellular carcinoma (HCC) interact with metabolism at the cellular and systemic levels [7]. The gut microbiota, through the gut–liver axis, significantly contributes to the development of HCC. Dysbiosis, as a consequence of a poor lifestyle, can be overcome by the use of probiotics and symbiotics. Metabolomics science and scientific technologies are reported to be crucial in detecting the biomarkers of liver cancer, and they are currently being considered the new tools to fight similar pathologies.

Molecular investigations, such as 16S rRNA sequencing, were used in a study concerning the variability of human microbiome in the nasopharyngeal site in a population at different ages [8]. The variability in the nasopharyngeal microbiome is associated with patient’s susceptibility to several infections, so it is thought that the nasopharynx may play an important role in health and disease.

The composition of the placental microbiome and the relative microbial characteristics were taken into account by Stupak et al. regarding their role in placental development and function in late fetal growth restriction (FGR) [9]. In this study, the microbiome of normal and FGR placentas were compared, and the bacteria present in both placentas were identified by an analysis of bacterial proteins set through proteomic and bioinformatic studies. The authors demonstrated that placental dysbiosis could be an important factor in the etiology of FGR, leading to the conclusion that placental microbiota and its metabolites may greatly affect the screening, prevention, diagnosis, and treatment of FGR.

A relationship between microbiome composition and the brain is often reported. Given that dysbiosis in the gut microbiota is involved in many neurodegenerative diseases such as Parkinson’s and Alzheimer’s, it could be assumed that Huntington’s disease (HD) can also be induced by dysbiosis, highlighting the essential role of the intestine–brain axis in HD pathogenesis and evolution [10].

The correlation between gut with lung microbiota, mast cells, platelets, and SARS-CoV-2 was also studied [11]. It was demonstrated that an altered condition of gut microbiota, especially in elderly patients, could be an important factor and have a strong impact on lung homeostasis and COVID-19 together with the activation of mast cells and platelets, and also influence the outcome of the pathology. Changes in the microbial population of elderly people can lead to a chronic state of inflammation, which affects host–microbiome interactions and increases the weaknesses of seniors.

The relationship between gut microbiota and *Clostridioides difficile* infection (CDI) has been widely studied by many researchers from different perspectives. In Piccioni A et al.’s study, it is underlined which types of microbiota alterations are most at risk for the onset of this infection [1]. CDI is one of the greatest public health challenges worldwide as its role is crucial to the interaction with the microbiota. Other than the classic antibiotic treatment, numerous therapeutic alternative strategies have been developed against CDI, such as the use of bacteriophages, fecal microbiota transplantation, both active and passive immunization, and products of the human microbiota that counteract the occurrence of *Clostridioides difficile* infection.

A very interesting topic is the possible impact of non-pathogenic bacteria on the spread of virulence and resistance genes among microbiomes [12]. The role of the above microorganisms is underlined and evaluated in the light of virulence, drug resistance, and other kind of genes able to increase the success of pathogens during infection. Commensal bacteria, including those with drug resistance or virulence genes, could colonize and be transmitted to susceptible hosts for prolonged periods by helping pathogenic cells to receive these genes, and consequently amplify their presence among microbiomes.

Definitely, the principal and crucial role of the gut microbiome includes its relationship with pain [13]. It is well known that the microbiome may lead to different pathologies through the gut–brain axis. The disorder of this relationship is not only associated with gastrointestinal syndromes but also with different types of disease such as cancer, neurological, and cardiovascular and metabolic diseases other than inflammatory disturbances and migraine attacks. Consequently, the human microbiome may be an essential component of the pathogenesis of multiple types of pain. Probiotics, a dietary restriction of short-chain fermentable carbohydrates (low-FODMAP diet), and fecal microbiota transplantation are reported to be beneficial for reducing symptoms and pain episodes.

In summary, the articles in this Special Issue provide a great range of reviews and updates to the role of gut microbial metabolites in the maintenance of health and homeostasis within the human body, as well as to the prevention of infection and disease. The potential anti-cancer properties of different groups of gut metabolites against various cancer types play a crucial role and should be further investigated (Figure 1). Therefore, understanding the specific link between the microbiome and each type of cancer is vital for developing effective treatments.

## Figures and Tables

**Figure 1:**
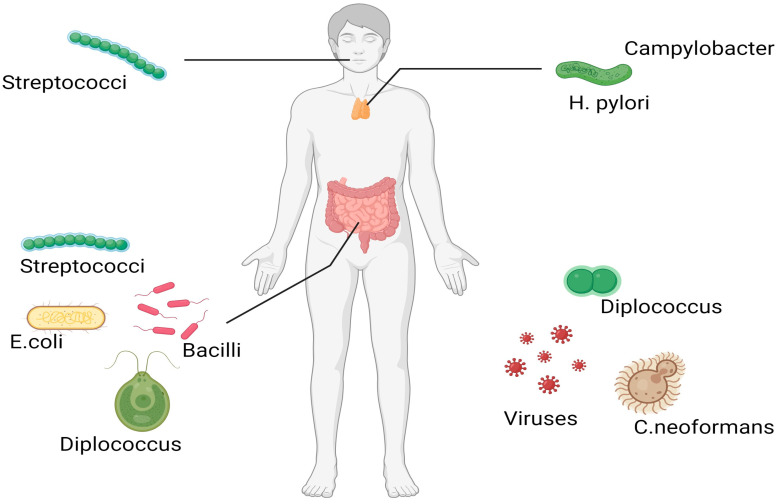
Human microbiomes in different parts of the body. These bacteria can affect cancer progression and the outcome of other diseases. Created with BioRender.com (accessed on 21 September 2023).
